# 766 12-year Analysis of Gender Disparity in Authorship of Peer-Reviewed Burn and Wound Care Literature

**DOI:** 10.1093/jbcr/irac012.319

**Published:** 2022-03-23

**Authors:** Iman F Khan, Suvethavarshini Ketheeswaran, Ismail Turker, Elvin Alasgarov, Brea Wiley, Gunel Guliyeva

**Affiliations:** Boston University School of Medicine, Baltimore, California; Johns Hopkins University School of Medicine, Baltimore, Maryland; Mayo Clinic Jacksonville, Jacksonville, Florida; Mayo Clinic Jacksonville, Jacksonville, Florida; University of Miami Miller School of Medicine, Miami, Florida; Johns Hopkins University School of Medicine, Baltimore, Maryland

## Abstract

**Introduction:**

The importance of gender equity and gender representation in academic publications has long been emphasized in medicine. It has been established that women represent a smaller proportion of primary and senior authors in high-impact medical journals than men and that original research articles written by women as primary and senior authors are less frequently sited than those authored by men. Currently, there is limited data evaluating whether this gender bias is present in plastic surgery and burn publications. We used bibliometric analysis of original research publications to analyze gender bias against women in one burn journal.

**Methods:**

Using the journal, Burns, we conducted a bibliometric analysis of research publications from 2009 to 2020. A gender determining application was used to characterize the gender of the first and senior author. Ratios of male:male, female:male, male:female, and female:female were obtained and analyzed.

**Results:**

Of the 1677 publications included, 40% have female first authors and 25.5% had female senior authors. Male:male authorships had the highest number of publications. Female:female authorship had the lowest number of publications of all the other ratios from 2009-2012, however there was a steep increase in 2013 in which male:female authorship had the lowest number of publications. Male senior authorship was associated with 2.9-fold increase in male first authorship [OR=2.99(95% CI 2.39, 3.76); p < 0.0001).

**Conclusions:**

Female representation in senior authorship positions in burn and wound care publications is increasing, however is still far from reaching gender parity. By analyzing authorship ratios by gender, we recommend a new way to evaluate gender disparity in burn and wound care academia.

